# A Pilot Study of Iyengar Yoga for Pediatric Obesity: Effects on Gait and Emotional Functioning

**DOI:** 10.3390/children5070092

**Published:** 2018-07-04

**Authors:** Keri R. Hainsworth, Xue Cheng Liu, Pippa M. Simpson, Ann M. Swartz, Nina Linneman, Susan T. Tran, Gustavo R. Medrano, Bryant Mascarenhas, Liyun Zhang, Steven J. Weisman

**Affiliations:** 1 Jane B. Pettit Pain and Headache Center, Department of Anesthesiology and Children’s Hospital of Wisconsin, Medical College of Wisconsin, Milwaukee, WI 53226, USA; sweisman@mcw.edu; 2 Department of Orthopedics, Children’s Hospital of Wisconsin, Medical College of Wisconsin, Milwaukee, WI 53226, USA; xliu@chw.org; 3 Department of Pediatrics, Medical College of Wisconsin, Milwaukee, WI 53226, USA; psimpson@mcw.edu; 4 Division of Quantitative Health Sciences, Medical College of Wisconsin, Milwaukee, WI 53226, USA; Liyzhang@mcw.edu; 5 Department of Kinesiology, University of Wisconsin-Milwaukee, Milwaukee, WI 53211, USA; aswartz@uwm.edu; 6 Department of Educational Psychology, University of Wisconsin-Milwaukee, Milwaukee, WI 53211, USA; Linnema7@uwm.edu; 7 Department of Psychology, DePaul University, Chicago, IL 60614, USA; susan.tran@depaul.edu; 8 Department of Psychiatry, University of Illinois at Chicago, Chicago, IL 60612, USA; gmedrano@uic.edu; 9 Santosh Yoga, LLC, Brookfield, WI 53005, USA; bryant@santoshyoga.com

**Keywords:** yoga, obesity, pediatrics, biomechanics, health-related quality of life

## Abstract

Obesity negatively impacts the kinematics and kinetics of the lower extremities in children and adolescents. Although yoga has the potential to provide several distinct benefits for children with obesity, this is the first study to examine the benefits of yoga for gait (primary outcome) in youths with obesity. Secondary outcomes included health-related quality of life (HRQoL), physical activity, and pain. Feasibility and acceptability were also assessed. Nine youths (11–17 years) participated in an eight-week Iyengar yoga intervention (bi-weekly 1-h classes). Gait, HRQOL (self and parent-proxy reports), and physical activity were assessed at baseline and post-yoga. Pain was self-reported at the beginning of each class. Significant improvements were found in multiple gait parameters, including hip, knee, and ankle motion and moments. Self-reported and parent-proxy reports of emotional functioning significantly improved. Time spent in physical activity and weight did not change. This study demonstrates that a relatively brief, non-invasive Iyengar yoga intervention can result in improved malalignment of the lower extremities during ambulation, as well as in clinically meaningful improvements in emotional functioning. This study extends current evidence that supports a role for yoga in pediatric obesity.

## 1. Introduction

The prevalence of obesity in pediatric populations has tripled in the past three decades [1,2]. It is alarming to consider that 90% of obese adolescents will remain obese at least into their thirties [3] and severe obesity (>99th percentile for gender and age) is the fastest-growing subcategory of pediatric obesity [4], particularly in children 2–5 years of age [5]. Obesity negatively affects numerous bodily systems [6] and multiple facets of health-related quality of life (HRQoL) [7,8]. In fact, the impact of severe obesity on HRQoL in pediatric samples has been reported to be similar to that of children undergoing chemotherapy for cancer [7]. Obesity in children and adolescents has also been identified as a risk factor for painful conditions, such as osteoarthritis [9,10], musculoskeletal pain [11], and headaches [12]. Although exercise is viewed as a critical component of obesity interventions [13], aberrant biomechanics in youth with obesity have raised concern over traditional exercise prescriptions [14]. This concern arises from apprehension about the structural integrity of joints over time, due to repeated high stresses, that occur even with walking [14,15,16]. While studies involving older adults have shown that yoga can improve gait biomechanics [17,18,19], and increasing evidence suggests that yoga may be a promising intervention for obesity in children [20] and adults [21], no studies have examined the benefits of yoga for gait biomechanics in children with or without obesity.

The need for interventions to improve gait in youth with obesity is underscored by the negative effects of excess weight on the joints and associated poor biomechanics [16,22,23,24,25,26]. Obesity-related biomechanical changes in youth may result in profound and lasting effects on musculoskeletal and joint health, particularly considering the impact over time and developmental stage [22,23,25,27,28]. As compared with normal weight peers, obesity in children significantly alters the force on the joint (i.e., the joint moment) and its power, which may result in musculoskeletal malalignment and the elimination of biomechanical function. These changes eventually result in pain, impaired ability to engage in physical activity, and decreased quality of life [22]. Due to the alterations in gait to accommodate the excess mass, children with obesity are more susceptible to certain musculoskeletal disorders, including anatomic destruction and functional limitation. Children with obesity commonly have a mechanical deficiency in the medial tibial growth plate which can result in the bowing of the tibia [23,28], known as tibia varus or Blount’s disease. Children with obesity can also experience slipped capital femoral epiphysis around skeletal maturity, where the femur is rotated externally from the growth plate causing pain and the inability to walk [22,29].

The extant literature suggests that yoga may be a promising intervention for obesity in children [20] and adults [21,30]. Specifically, yoga (1) may increase strength, flexibility and stamina [17,20,31], (2) has been shown to improve multiple domains of HRQoL [32], (3) may be a stepping-stone to increased physical activity [20,33], and (4) is beneficial for those with existing pain conditions [34]. Finally, although only studied in elderly adults, yoga has been shown to improve function and strength in the lower extremities. For example, DiBenedetto et al. [19] found increased peak hip extension and stride length after eight weeks of Hatha yoga. Zettergren et al. [18] found improved balance and increased fast walking speed after an eight-week Kripalu yoga intervention. More recently, Wang et al. [17] found improvements in lower extremity muscles (e.g., isometric knee flexor strength) after a 32-week Hatha yoga intervention modified for senior adults.

While many types of yoga exist, the Iyengar style has distinct benefits for youth with obesity, particularly for those with co-morbid pain conditions. The Iyengar style is characterized by the use of “props” (e.g., mats, blankets, blocks) that increase stabilization and allow for a slow and safe practice of the postures [35]. This style is also characterized by “… the emphasis on (…) protecting the joints, promoting circulation, and compensating for limitations in strength, flexibility, and mobility” [36] (p. 66). Instructions are given throughout classes, and are focused on awareness of muscle and joint activity [37,38,39]. Poses are typically “held” for 30–60 s, however, poses can be tailored to the individual weight-related or pain-related needs of the participant [20,39]. These aspects of Iyengar yoga are particularly important when considering the obesity-related mechanical forces on the lower back and lower extremities and the negative effect of obesity on balance [25,40,41,42,43].

The purpose of this pilot study was to extend our original study of yoga for pediatric obesity [20] to include gait biomechanics as an objective assessment of benefits associated with yoga. The primary aim was to determine whether an eight-week Iyengar yoga intervention would improve gait biomechanics (kinematics and kinetics) in youth with obesity. Secondary aims were to examine feasibility, acceptability, and participant/parent perspectives about the yoga intervention, and whether the intervention would improve HRQoL (as reported by participants and their parents) and pain in participants with co-morbid pain complaints. We also sought to explore whether participants would show pre-to-post yoga increases in physical activity.

## 2. Materials and Methods

### 2.1. Overview

The study utilized a within-participants pre-post experimental design centered on an eight-week Iyengar yoga intervention. Demographics (gender, age, and race) were extracted from the electronic medical record. Outcomes assessed before and after the intervention included: height and weight, assessments of yoga expectations and past experiences with yoga (pre-intervention only) and perceptions of the benefits of yoga (post-intervention only), health-related quality of life, physical activity, and gait analysis. Height, weight, and gait analyses were conducted no more than two weeks before the first yoga class; post-yoga gait analyses were conducted no more than one week after the final yoga class. All questionnaires were completed immediately prior to the start of the first class (pre-yoga) or immediately after the last class ended (post-yoga). Current pain intensity was reported immediately before the start of every yoga class. This study was approved by the Children’s Hospital of Wisconsin Human Research and Review Board, number CHW 11/121 (approved on 20 July 2011).

### 2.2. Participants

Youths 11–18 years of age were either referred by their primary care physician or were recruited from the Nutrition Exercise and Weight (NEW Kids) clinic at the Children’s Hospital of Wisconsin. Referral to the NEW Kids Program requires that patients have a body mass index (BMI) ≥ the 95th percentile, and at least one medical comorbidity [44]. The study was explained to all participants by the first author, who also obtained parental consent and participant assent. All participants received medical approval to participate in the study by their primary care physician. Exclusion criteria included the following: past spinal injuries or surgery, neck pain associated with an injury, medical practitioner’s non-approval for any reason, any limitations to walking (e.g., broken ankle, wheelchair bound), cognitive impairment, developmental delay, and non-English speaking.

Participants were provided with gift card incentives to compensate for the time needed to complete the questionnaires and gait analyses, and for the return of the Actigraph accelerometer (Actigraph, LLC, Pensacola, FL, USA). Parents were provided with a gas card after the 4th and 16th classes.

### 2.3. Procedure

Yoga Intervention. The intervention consisted of bi-weekly, 60-min classes, for eight weeks (16 total classes). The intervention was designed and classes were taught by an experienced yoga practitioner (BM), a certified toga teacher (CYT), expert-registered yoga teacher (E-RYT), and Kripa Foundation Iyengar Yoga (KFIY) practitioner with 26 years of teaching experience. The core of the intervention involved yoga poses (*asanas*) and controlled breathing (*pranayama*) consistent with the Iyengar style of yoga, and based on the principles of B.K.S. Iyengar [35,39]. Participants were given all equipment needed for the yoga classes to use free of charge during the intervention. Equipment included a mat, 2–3 m straps, 3 blankets, and 2 wooden blocks. The yoga classes were held in a large conference room located in a satellite clinic.

The yoga curriculum is shown in Table 1. The curriculum was designed to focus primarily on the physicality of yoga practice. Asanas were chosen specifically to strengthen and align the trunk and lower extremities. Classes included instruction/demonstration of each asana, followed by participant practice. Participants reported current pain intensity at the beginning of each class. The instructor actively modified asanas where necessary for pain and/or other limitations (e.g., body habitus), and gave instruction on breathing technique throughout. Consistent with Iyengar teachings, all asanas focused on proper alignment (actions and movements). A novel aspect of the intervention was the inclusion of a Walking Tadasana. While the movement aspect of this pose is not typical of Iyengar yoga, all other aspects of this pose are consistent with the Iyengar Standing (static) Tadasana. For this pose, participants were instructed to walk the length of the room 3–4 times, during which, they would focus their attention on three aspects of their posture: (1) standing up straight, (2) lifting the pit of their abdomen, and pointing their toes forward.

### 2.4. Primary Outcomes

Gait Assessment. Within two weeks before the intervention began, and within a week after it ended, all participants underwent a gait analysis. Before each gait analysis, anthropometric measurements were taken for the computer model input, including weight (kg), height (cm), bilateral knee (cm) and ankle (cm) diameters, leg lengths (cm), and inter ASIS (anterior superior iliac spine) distance (cm). A lower limb Plug-in-Gait marker set and body model was used in this study (Vicon Systems, Oxford, UK). The reflective markers are placed on the skin, and the set is composed of the bilateral ASIS (2), PSIS (posterior superior iliac spine) (2), thighs (2), knees (2), shanks (2), ankles (2), heels (2), and 2nd metatarsal heads (2). Due to the increased amount of adipose tissue in the abdomen area, it is important to place the markers as close as possible to firm bony landmarks, while still being visible to the cameras. If the anterior pelvis were obscured, the markers were moved 1 cm laterally as recommended by the Plug-in Gait model. Using this method, the maximum deviation in any joint at any plane is 5.25 degrees, which is found at the sagittal plane of the knee [45]. On average, there is a 2.88-degree deviation from actual measurements [45]. 

Kinematic data were captured with twelve T-40S cameras using the Vicon motion capture system (Vicon Systems, Oxford, UK). Ground reaction force data was measured through four force plates (Bertec Corp, Columbus, OH and AMTI, Watertown, MA, USA) which is integrated with the Vicon motion capture system. Reverse kinetics were also calculated through Vicon’s motion capture system, which calculates the ankle, knee, and hip moments. 

All participants began the gait assessment with a static calibration trial. During the static trial the knee markers are replaced with knee alignment devices (KAD), which were composed of three of its own reflective markers. The KADs provided a separate calculation of the orientation of the mediolateral axes of the knee and ankle. The participants were also asked to stand in a straight, upright posture. Overall, the static trial reoriented the virtual ASIS markers by using the physical examination measurements, and standardized the orientation planes. 

After the static trial, The KADs were replaced by reflective markers on the lateral knee cap for the dynamic trials. The patients are asked to walk barefooted along a 30-foot walkway at a self-selected pace. Ten trials for each patient are recorded. Raw data are filtered through a Butterworth digital filter (15 Hz cutoff frequency). Three trials with minimal noise were chosen for further processing; they were standardized to a single gait trial through Vicon Polygon (Vicon Systems), and averaged afterwards.

The stance phase was divided into Loading Response (0–17%), Mid-Stance (17–50%), Terminal Stance (50–83%), and Pre-Swing (83–100%) phases. The swing phase was divided to Initial Swing (0–33%), Mid-Swing (33–68%), and Terminal Swing (68–100%). Maximum and minimum kinematic (entire stance and swing phases) and kinetic (entire stance phase) were measured for each patient. Additionally, peak kinematic parameters are measured for all sub phases in the gait cycle, while peak kinetic data was measured for all sub phases in the stance phase.

### 2.5. Secondary Outcomes

Feasibility and acceptability were based on results from our previous study of yoga for obese youth [20]. For the intervention to be deemed feasible, at least 50% of those recruited would be “completers” of the intervention, having attended at least 1/3 of the total number of classes (i.e., at least five classes). Details on recruitment were used to complement the attendance rate data. To be deemed acceptable, participant and parent ratings of perceived yoga benefits would be positive (defined as a median of 5.0 or higher on all likert-based questions in the Holistic Health Questionnaire; see below).

The Holistic Health Questionnaire (HHQ; participant and parent report) was used to assess acceptability, as well as to capture participant and parent perspectives on the yoga intervention. This brief measure was adapted from the HHQ originally developed by Zeltzer et al. [46]. It has been used to understand patient and parent perspectives on complementary and integrative approaches to health [20,46]. For this study, respondents were asked about past experience with yoga and expectations of the yoga intervention. Questions included expectations about yoga’s value for their (their child’s) pain relief and sleep, and expected pleasantness of yoga. Responses were rated on 7-point Likert scales (e.g., 0 = will not help, 7 = will definitely help), with higher numbers indicating positive expectations. Space was provided for written comments focused on general expectations of yoga that did not include pain relief. Post intervention questions included ratings of the value of the current yoga intervention for pain (if applicable) and sleep, and the pleasantness of the yoga experience. Space was provided for written comments. 

Health-related quality of life. The Pediatric Quality of Life Inventory, v.4.0 (PedsQL 4.0; participant and parent report) [47] was used to assess HRQoL. The PedsQL is a brief, 23-item self-report used to assess HRQoL. Age appropriate versions were completed as self-reports and as parent-proxy reports. Health-related quality of life is measured in four domains, including physical functioning (eight items), emotional functioning (five items), social functioning (five items), and school functioning (five items). Based on a 5-point Likert scale, respondents are asked to indicate how much of a problem each item has been during the past month. Responses ranged from 0 (never a problem) to 4 (almost always a problem). Ratings for each area of functioning were reverse scored, transformed to a 0–100 scale (0–100, 1 = 75, 2 = 50, 3 = 25, 4 = 0), and averaged. Lower scores indicated a lower HRQoL. The PedsQL yielded a score for each subscale, a Physical Health and Psychosocial Health Summary Score, and a total quality of life score. The Physical Health Summary Score was the same as the score for the Physical Health Subscale, whereas the Psychosocial Health Summary Score, was the average of the emotional, social, and school functioning subscales. The PedsQL has shown excellent reliability and validity [48]. Reliability for the total score in this sample was excellent for both parent proxy report (Cronbach’s α = 0.88) and participant self-report (Cronbach’s α = 0.84).

Pain Intensity. Pain intensity was reported at the start of every class. Pain intensity was rated on a numeric rating scale (NRS), with 0 = no pain and 10 = worst pain. Numeric rating scale use has been validated in pediatric pain populations [49]. 

Physical Activity was measured with a tri-axial ACC (Actigraph GT3x, ActiGraph, LLC), worn on an elastic belt, and situated above the right hip. The Actigraph provides continuous monitoring of the participant’s activity levels. Participants were given verbal and written instructions on the correct placement and wear of the activity monitor. Participants were asked to wear the activity monitor for seven continuous days both before and immediately after the yoga intervention (14 days total), and to be worn all waking hours, except when bathing or swimming. Prior to beginning the yoga intervention, the devices were mailed to participants 2–3 days before the start of the 7-day pre-yoga period, and were returned before beginning the first yoga class. For the post-yoga activity monitoring, devices were given to participants after the last yoga class, and were mailed back after the seven day period using a postage-paid envelope.

### 2.6. Data Analyses

Descriptive statistics were utilized to characterize the sample. Means and standard deviation (SD) were presented for normally distributed data, and where skewed, data were presented as medians and interquartile range (IQR). Acceptability of the intervention was based on attendance, and quantitative and qualitative assessments of the benefits associated with the yoga intervention, as reported by participants and parents. Paired Wilcoxon tests were conducted to determine pre- to post-yoga changes in all outcomes. Cohen’s *d* was calculated for significant pre-post yoga changes. IBM SPSS v 22 (IBM Corp., Armonk, NY, USA) was used to conduct analyses.

Accelerometer data were downloaded in 1 s epochs using Actilife v6.13.3 (ActiGraph, LLC). Wear-time was validated using the Choi algorithm. Participants were included in analysis if the device is worn for a minimum of 10 h per day on four days. Time spent in physical activity intensities were calculated using cut points developed by Evenson et al. [50] to determine time spent performing sedentary behavior, and light-, moderate- and vigorous-intensity physical activity.

## 3. Results

### 3.1. Participants

Ten participants were enrolled in the study, however, one of the participants only attended the first three of the 16 classes, and was therefore excluded from all analyses. The median age of the nine participants who completed the study was 14 years (range 11–17 years). Five participants were female, five self-identified as Caucasian, two Hispanic, one African-American, and one Asian. Pre-yoga, all participants’ BMI was ≥ the 96th percentile for gender and age [51]; six of nine had severe obesity, with a BMI >99th percentile [5]. As expected, from pre- to post-yoga, participants’ BMI percentile was exactly the same, with a median of 99, and IQR 98–99 (*p* > 0.05). None of the participants had any prior experience with yoga. 

### 3.2. Gait

Significant pre- to post-yoga changes in joint motions at the hip, knee, and ankle are shown in Table 2. There was significantly increased hip adduction and reduced hip abduction in both the Stance and Swing phases (*p* < 0.05). There was significantly increased knee valgus at heel contact but reduced knee varus during Stance phase and Initial Swing phase (*p* < 0.05). The ankle joint presented with a significant increase of plantarflexion but reduced dorsiflexion during swing phase.

Also shown in Table 2 are significant pre- to post-yoga changes in joint moments. The hip internal rotation moment (Nm/kg) in heel contact in stance was increased from a median of 0.02 to 0.03 Nm/kg (*p* = 0.028). The maximum adduction moment (Nm/kg) in stance phase was reduced from a median of 0.12 to 0.09 Nm/kg (*p* = 0.028). With regard to temporal parameters (not shown in table), cadence was significantly reduced from pre- (119.2, 109.8–123.3 steps/minute) to post-yoga (117.7, 104.7–118.6 steps/minute). Similarly, velocity was significantly reduced from pre- (1.1, 1.0–1.2 m/second) to post-yoga (1.0, 1.0–1.2 m/second) (*p* = 0.013). However, stride length did not change from pre- (1.1, 1.1–1.2 m) to post-yoga (1.1, 1.1–1.2 m) (*p* = 0.11).

Overall, the pattern of results shows reduced malalignment during walking, particularly at the hip and knee joints.

### 3.3. Feasibility and Acceptability

Based on class attendance, the yoga intervention is feasible. Nine of ten participants were considered “completers”. Three of the nine participants attended 10–11 of 16 classes (63–69%), and five attended 13–16 classes (81–100%). One attended only the first three classes and was excluded from analysis.

Recruitment initially began in the weight management clinic, however, this method was insufficient. In total, four were recruited using this method over a period of 2.5 months, due primarily to a high no-show rate in the clinic. Of these four approached, all consented, but one withdrew before the intervention began, due to loss of transportation. Recruitment by referral from pediatricians in the local area was more successful. In total, 17 patients were referred by their pediatricians over a period of 2.5 months. Of these, a total of seven consented. Of the ten who were referred but did not consent, three never responded to the initial phone call, one could not participate because he was currently in physical therapy, one did not receive medical clearance by her orthopedist due to patellofemoral syndrome, the parent of two patients declined after hearing about the study because she did not think her sons would like yoga, and another declined because the class time would not work for the family. Although two siblings referred initially expressed interest in the study, they did not show up for their consent/gait appointment.

Based on responses to the quantitative questions on the HHQ (see Table 3), the yoga intervention is acceptable. Final HHQ questionnaires were completed by nine of nine participants and eight of the nine parents. Quantitative responses are shown in Table 3 and qualitative responses in Table 4. Overall, responses from both participants and parents were positive. Most evaluations of the experience by the participants, or by parents (based on their perception of benefits for their child), included ratings of 5–7 (7 = highest rating). Participants and parents reported a number of physical (e.g., “It helped a lot with my ankles” and “flexible and run faster”) and psychosocial (e.g., “The best thing I got from this study is that I feel better about myself”) benefits.

### 3.4. Health-Related Quality of Life

Participants reported significantly improved overall HRQoL, as well as improved psychosocial functioning, with both scores primarily attributable to improvements in emotional functioning. Parent-proxy reports of emotional functioning also improved significantly from pre- to post-yoga. Scores are shown in Table 5. For all scores with significant pre- to post-yoga changes, there was a medium effect size (Cohen’s *d*), and the median change score represents a clinically meaningful improvement [48,52].

### 3.5. Pain Intensity

Analyses of changes in pain intensity across time were precluded by the sample size and number of participants who reported pain. Pre-intervention, three of none participants reported moderate-severe (a score of 4–6 on a NRS) pain, including pain complaints of chronic low back pain, chronic knee pain, and recurrent headache pain. Despite these pain complaints, these three participants attended 11, 10, and 16 classes, respectively. To provide more details about patients with obesity and comorbid pain in the context of a yoga intervention, three case examples are included.

Case #1. A 17-year-old male with chronic pain in the lower back, and a BMI at the 99th percentile. His mother reported that he went to bed with pain of 7/10 on a nightly basis. Before the yoga intervention began, he indicated that he did not have expectations about what yoga could do for him, but his mother expected yoga to reduce his stress. Before the first class, he reported a current pain intensity of 5/10. He attended 11/16 classes. Of the nine completers, he reported pain most consistently, with pain intensity ranging from 2–5, and pain reported on 8 of the 11 classes attended. Of note, his self-report of physical functioning worsened over the course of the intervention, with a change score of −15.6. However, his self-reported emotional functioning improved, with a change score of 15.0. His mother reported that he improved physical functioning (change score of 9.4) and emotional functioning (change score of 10.0). In the qualitative comments, his mother expressed that she hoped her son learned how yoga could help with stress and anxiety. 

Case #2. A 12-year-old male with chronic knee pain, and a BMI above the 99th percentile. He expressed no expectations for yoga, however, his parent hoped that the following might occur: “increased core strength, flexibility, confidence, and possibly weight loss.” Before the first class, he reported a current pain intensity of 6/10, however he reported having no pain for the remainder of the intervention. Of all participants, he and his parent reported the greatest improvements across all domains of HRQoL. Specifically, his reported physical functioning improved from 62.5 to 81.3 (change score of 18.8), and emotional functioning improved from 70.0 to 85.0 (change score of 15.0). His parent’s proxy change scores were similar, with a change score for physical functioning of 31.3, and emotional functioning of 15.0. By the third class, this participant began to report improvements in the way he felt, and behaviorally, he began to tuck in his shirt. Post-yoga, his parent reported that he had lost weight, and was “very motivated in keeping up with his health.” Both participant and parent expressed interest in continuing yoga classes together in the future. 

Case #3. A 16-year-old female with intermittent musculoskeletal pain, and a BMI at the 96th percentile. Before the intervention, her parent expressed hope that the yoga would help her with her weight and “help her with her attitude.” She did not have any expectations. The only pain she reported throughout the intervention was before the 10th (5/10), 11th (10/10) and 12th (5/10) classes. On the day of the 11th class, her pain flared to a 10/10. Although she attended the class, she did not want to participate. However, the instructor modified all poses for her during the class, which enabled her to engage. When the class was finished, although her pain report was a 9/10, she reported that her pain was “50% better.” Both she and her parent reported improved physical functioning (both change scores of 12.5) and improved emotional functioning (change scores: self-report 10.0, parent-proxy report 5.0). She reported that she felt “less tired” after the yoga intervention.

### 3.6. Physical Activity

Of the nine completers, five had reliable accelerometry data. Average daily accelerometer wear time was 14.8 h pre-yoga and 15.0 h post-yoga. Participant activity was primarily sedentary, with about an average of 11 h (approximately 84–86% of the day) pre- and post-yoga spent in sedentary pursuits. This equates to approximately 67% (pre) and 66% (post) of total daily wear time (data shown in Table 6). The amount of time spent in varying levels of physical activity did not change from pre- to post-yoga (all *p*’s > 0.05).

## 4. Discussion

While the literature suggests that yoga may provide several distinct benefits for adults and children with obesity, no previous studies have examined the benefits of yoga for gait in any pediatric population. In this study, children and adolescents with obesity participated in an eight-week Iyengar yoga intervention. A number of significant improvements in gait were observed, reflecting reduced malalignment of the lower extremities and increased strength of the hip flexors and adductors during ambulation, especially at the knee and hip joints. Additionally, participant- and parent-proxy reports indicated significantly improved emotional functioning. Although recruitment posed challenges not unlike our previous pediatric yoga trials [20,53], this intervention was acceptable to participants, including those with chronic and recurrent pain conditions. These results are consistent with our previous studies, and suggest that yoga has the potential to play an important role in pediatric obesity.

Children with obesity typically have poor biomechanics and an altered gait that puts them at risk for musculoskeletal malalignment, pain, and injury over time [16,22,23,26,41]. One such example includes wider strides during ambulation, which can result in an increased base support, and is done to compensate for a loss of balance. While research attention has been given to understanding how and why obesity impairs locomotor function, ways to improve function has received little attention. Some have observed improved gait in adults, after bariatric surgery-induced weight-loss. For example, a recent review of the literature [54] indicates that weight loss after bariatric surgery is associated with increased swing time, stride length, gait speed, and lower extremity range of motion. In the pediatric literature, an intervention was designed specifically to improve biomechanics during walking and running in youth with obesity. Steinberg et al. [55] showed that after a six-month multidisciplinary obesity management intervention (which included dietary and exercise components), youth with obesity had improved foot pressure during walking and running at different speeds. The exercise component was specifically focused on improving locomotion, and included exercises such as skipping rope and jumping on different surfaces. In a different study, Delextrat et al. [56] found improved biomechanics after adolescents participated in a weight loss intervention which included three 60-min sessions per week, for a period of eight weeks. In addition to other benefits, the authors found that participants’ walking speed increased by 23% and the energy cost of walking at different frequencies was decreased. In the context of these findings, several points about our Iyengar yoga intervention are worth noting. First, in the current study, participants achieved a number of gait improvements, in a relatively short period of time, and at a relatively low dosage. In contrast to these more involved and time-consuming interventions, our improvements were seen after only ≤16 classes over an eight-week period. Second, unlike surgery, the yoga intervention in this study was non-invasive, and therefore avoids all of the risks inherent in surgical interventions. It is also a life-long activity that provides a number of physical and psychosocial benefits. Finally, although the dietary and exercise interventions reported in the literature resulted in improved outcomes for participants, some of the included exercises would be contraindicated for some obese participants, particularly those with comorbid pain conditions. For example, our participant in Case #1 would not have been able to jump rope or jump on different surfaces. In contrast, the Iyengar yoga poses were modified on an as-needed basis, which allowed participants to engage in the classes despite factors that may have precluded their involvement in any other physical activity intervention. Importantly, the slow, gentle movements in Iyengar yoga and the modification of poses to meet individual needs reduce the risk of new injuries and the risk of exacerbating existing pain conditions. 

The pattern of gait changes observed in this study suggests improved balance and overall reduced malalignment of the lower extremities, and increased strength of hip muscles while walking. These specific functional improvements cannot be attributed to changes in body weight or physical activity, as both remained stable for participants across the intervention. Rather, consistent with the literature on yoga [17,57,58], we hypothesize that the gait changes were primarily due to increased muscle strength and flexibility as a result of the intervention. For example, the increased hip internal rotation moment implies that the yoga intervention resulted in increased strength of the hip flexors and/or adductors. In contrast to the overall improvements observed however, we did find a reduction in velocity from pre- to post-yoga, and increases in walking speed are almost always viewed as an improvement. For example, Delextrat et al. [56] found a large and significant increase in gait velocity after their weight loss intervention for youth with obesity. However, increased velocity actually increases the force on the knee [59], which could potentially put these youth at greater risk of injury. The reduction in gait velocity that was observed in this study may therefore be beneficial for this population. Although this would need to be substantiated in future studies, we hypothesize that the reduced velocity may be attributable to the inclusion of the Walking Tadasana in our intervention. Specifically, it may be that the instruction and continuous practice of this asana, resulted in increased mindfulness about posture and lower extremity alignment while walking, which in turn, may have translated into a slower gait. 

While the impact of pediatric obesity on physical functioning is robust [7,8,60], studies have also shown that obesity significantly impairs emotional functioning [60]. In fact, as Riazi et al. point out [60], it is important for clinicians to be aware of obesity’s impact on the emotional and psychological domains because these areas are likely affected before obesity affects the physical domain. It is therefore noteworthy that our eight-week intervention significantly improved emotional functioning by self- and parent-proxy reports, with medium effect sizes. Although this improvement may have been mediated by the improvements in biomechanical functioning, it is likely that this outcome is a result of multiple factors. In a recent qualitative study on factors associated with yoga that promote weight loss, the authors found that yoga offers a number of behavioral, physical, and psychosocial elements that would be beneficial to those attempting to lose weight 61]. While it is important to clarify that weight loss was never mentioned to participants, nor did the research team expect participants to lose weight, the benefits identified by participants in the Ross et al. [61] study could have been part of the experience of our participants. For example, respondents in the Ross et al. study discussed the benefits of role modeling, acceptance and social support that were part of their yoga experience. It is plausible that our participants also benefited emotionally from a sense of community, acceptance and role modeling by others in the study and by the yoga practitioner. 

An important aspect of this pilot study was the assessment of feasibility and acceptability. First, based on attendance alone, we reason that the yoga intervention is feasible. However, as with our previous pediatric yoga trials [20,53], we experienced challenges recruiting for the eight-week trial, and as with these previous studies, the biggest challenge was busy family schedules. We have previously suggested ways to improve recruitment, and this study extends that list to include referrals from primary care physicians. It is difficult for families to attend bi-weekly classes for eight weeks, but referral by their physician increased our recruitment numbers. Future studies should examine this method to determine if it would increase success. Finally, although we had previously investigated the acceptability of a Hatha yoga intervention for youth with obesity, due to the changes to the intervention, we felt that it was important to reexamine acceptability. All participants and parents in the current study rated the pleasantness of the experience highly, and indicated that the intervention improved sleep, movement and pain. Particularly important to both feasibility and acceptability is the high attendance rate by almost all (9 of 10) participants in this study, including those with chronic and recurrent pain conditions.

Based on the results of this study, future research on the benefits of yoga for youth with obesity is warranted. While our study now adds to the larger body of research on the benefits of yoga for those with obesity, it is important to note that the vast majority of this work has focused on adults, particularly older adults. This is a missed opportunity to positively impact youth with obesity. Intervening while children are young has the potential to prevent years of physical and emotional disability that are well-documented in adults (e.g., see [62]). Interventions, however, must be carefully designed so as to reduce the risk of injury, as well as the risk of exacerbating existing pain conditions. One area that could be very promising for youth with obesity, is to develop tailored interventions that meet the specific needs of this population. For example, Wang et al. [17,57,58] have examined the “biomechanic profiles” of specific yoga poses. With this knowledge, they indicate that it may be possible to develop evidence-based yoga interventions, with the goals of “targeting specific joints or muscle groups, addressing specific deficits in strength and muscular endurance, promoting improvements in physical function (e.g., balance), or unloading pathological tissues and structures at risk of injury [57]. For example, Wang et al. [58] found that while a modified chair and downward facing dog pose strengthened muscles in older adults, these poses had different biomechanical effects. While both poses strengthened the hip and ankle extensor muscles, the modified chair pose was more effective in strengthening the quadriceps muscle, and placed less stress on the knee joint. This type of information could be very beneficial to the development of interventions for youth with obesity.

### Study Strengths and Limitations

This study utilized a rigorous, objective method to examine gait (kinetics and kinematics) in our participants. Additionally, our use of the Actigraph to measure physical activity is a strength, as accelerometry is the gold standard for the objective assessment of physical activity. The yoga curriculum was designed, and classes taught by a yoga instructor (B.M.) with the highest level of training (CYT, E-RYT). Study results are limited by the small sample size. Additionally, the HHQ is not validated. While a pre- post-design reduces variability in measures, the lack of a control group is also a limitation. Future studies should replicate these findings with a larger sample, perhaps best achieved with multiple waves over time or with a multi-site trial. Additionally, a randomized controlled design would allow causal determinations to be made, along with an examination of mechanisms underlying the benefits demonstrated in this study. Additional qualitative elements would also allow a better understanding of the yoga experience for this population.

## 5. Conclusions

Overall, the results of this study suggest that positive changes in gait are achievable with a relatively brief, non-invasive intervention, and as an added benefit, this intervention can result in improved emotional functioning. After an eight-week Iyengar yoga intervention, children and adolescents with obesity were able to walk more efficiently, with reduced abnormal alignment of the lower limbs, have less impaired mobility, and better balance. Better understanding of activities that improve the daily functioning of obese youth may allow for the development of effective interventions.

## Figures and Tables

**Table 1:** Yoga curriculum.

Approximate Duration	Asana ^1^
5 min	Warm-up
45–50 min	Supta Baddha Konasana with blankets—Reclined bound angle pose
	Walking Tadasana—Walking mountain pose ^3^
	Tadasana—Mountain pose
	Vrksasana—Tree pose
	Virabhadrasana II—Warrior pose
	Trikonasana—Triangle pose
	Upavistha Konasana—Seated wide angle pose
	Ardha Padmasana variation(s)—Half lotus variation
	Yoga Sthilasana—Yoga for the feet series
	Adho Mukha Svanasana—Downward Facing Dog
	Vanarasana—Monkey pose ^2^
	Parsvakonasana—Side anlge pose ^2^
	Uttihita Hasta Padangusthasana I—Standing extended hand foot to toe pose
5–10 min	Savasana—Corpse pose

^1^ Not always in the order shown, and all poses not used every class. ^2^ Poses added to the second half of the intervention. ^3^ Included each class to check structural alignment and to improve posture while walking.

**Table 2:** Significant kinetic and kinematic differences of the lower extremities before and after yoga therapy (*n* = 9, median (IQR), *p* < 0.05).

Parameter	Median (IQR)		*p-*Value
	Pre-Yoga	Post-Yoga	
**Hip Motion in the Coronal Plane (Degrees)**			
Maximum adduction in stance	9.7 (8.9, 10.8)	12.5 (11.7, 14.3)	0.028
Minimum abduction in stance	−2.6 (−6.4, −1.9)	−1.1 (−2.3, 2.1)	0.021
Abduction initial swing	−4.6 (−7.0, −2.9)	−1.5 (−3.9, 1.0)	0.021
Maximum adduction in swing	3.0 (2.8, 3.9)	5.9 (4.0, 9.1)	0.038
Minimum abduction in swing	−6.5 (−8.2, −3.8)	−1.7 (−4.9, −1.5)	0.017
**Knee Motion in the Coronal Plane (Degrees)**			
Valgus in heel contact in stance	−6.6 (−8.2, −5.8)	−10.0 (−12.3, −6.8)	0.028
Maximum varus in stance	3.8 (3.1, 7.4)	−0.6 (−3.2, 2.0)	0.008
Varus in initial swing	7.0 (5.4, 13.0)	1.9 (−1.0, 3.5)	0.008
**Ankle Motion in the Sagittal Plane (Degrees)**			
Minimum plantarflexion in swing	−10.2 (−12.7, −10.0)	−12.3 (−20.0, −9.9)	0.028
Maximum dorsiflexion in swing	6.1 (5.0, 6.5)	4.7 (2.9, 5.4)	0.038
**Hip Moment in the Transverse Plane (Nm/kg)**			
Maximum internal rotation moment in heel contact in stance	0.02 (0.02, 0.03)	0.03 (0.02, 0.04)	0.028
**Ankle Moment in the Coronal Plane (Nm/kg)**			
Maximum adduction moment in stance	0.12 (0.11, 0.13)	0.09 (0.05, 0.09)	0.028

IQR, interquartile range.

**Table 3:** Participant and parent responses to questions about the yoga experience.

Question ^1^	Participant Median (IQR)	Parent/Caregiver Median (IQR)
How much do you think yoga helped with your (your child’s) pain?	5.0 (5.0, 6.5)	6.0 (5.0, 7.0)
How much do you think yoga helped with your (your child’s) sleep?	5.0 (3.5, 7.0)	6.0 (4.3, 6.8)
How pleasant was this experience for you (your child)?	7.0 (5.5, 7.0)	7.0 (6.3, 7.0)
How much do you think yoga helped you (your child) feel better when moving?	6.0 (4.5, 7.0)	6.0 (5.0, 7.0)

^1^ Scale: 0 = “not at all” or “extremely unpleasant” to 7 = “definitely helped” or “extremely pleasant”; the Holistic Health Questionnaire (HHQ) is available upon request.

**Table 4:** Qualitative responses from participants and parents about the yoga experience.

Question	Participant	Parent/Caregiver
Other than potential effects on your current pain symptoms, what else do you think occurred as a result of the yoga sessions?	“It helped a lot with my ankles.” “I feel less tired” “Flexible and run faster” “Meet new people” “I have got more flexible.” “I feel lighter and more relaxed. I don’t get as tired during the day.” “The best thing I got from this study is that I feel better about myself”	“I find that she seems more active at home and more willing to help around the house. Mood has improved overall ... Since the injury at school she has done some stretching at home to try and improve her flexibility.” “I think (participant name) gained a good understanding of what yoga can do for you.” “Hopefully (participant name) learned how yoga can help with stress and anxiety.” “Her attitude has improved a lot.” “Proud of herself and how more flexible she has become. She actually has left class and said “That was fun.” Not a normal reaction after exercise!” “He lost 11 lbs as of 6 days ago and is very motivated in keeping up with his health. We are hoping to get him involved with more yoga in the near future.” ^1^

^1^ Despite this comment by the parent, this participant did not lose weight across the eight-weeks yoga intervention.

**Table 5:** Median (IQR) self- and parent-reported HRQoL scores pre- and post-yoga intervention.

	Pre-Yoga Intervention	Post-Yoga Intervention	Median Change	*p-*Value	Effect Size
**Self Report**					
Total score	82.6	88.0	5.4	0.024	−0.53
	(69.6, 89.7)	(76.6, 94.5)	(1.0, 13.0)		
Physical functioning	81.2	93.7	3.1	0.174	
	(73.4, 90.6)	(79.7, 96.9)	(−1.6, 17.2)		
Psychosocial functioning	80.0	88.3	5.0	0.007	−0.63
	(70.0, 90.8)	(75.0, 94.0)	(3.3, 11.7)		
Emotional	70.0	85.0	10.0	0.024	−0.53
	(57.5, 95.0)	(75.0, 94.0)	(−3.1, 12.5)		
Social	90.0	93.7	10.0	0.103	
	(72.5, 97.5)	(82.50, 100.0)	(0.00, 17.5)		
School	80.0	90.0	10.0	0.072	
	(70.0, 80.0)	(75.0, 90.0)	(2.5, 15.0)		
**Parent Report**					
Total score	82.6	82.1	3.3	0.397	
	(62.0, 88.6)	(67.7, 90.2)	(−4.08, 14.2)		
Physical functioning	75.0	82.8	10.9	0.062	
	(65.6, 85.9)	(75.8, 96.9)	(−2.3, 21.1)		
Psychosocial functioning	83.3	80.0	−0.83	0.866	
	(60.0, 91.7)	(66.7, 87.9)	(−6.7, 10.4)		
Emotional	70.0	77.5	7.5	0.011	−0.64
	(52.5, 87.5)	(65.0, 88.7)	(5.0, 13.7)		
Social	95.0	82.5	−2.5	0.461	
	(60.0, 100.0)	(56.2, 97.5)	(−10.0, 0.0)		
School	80.00	72.50	−3.75	0.598	
	(57.5, 97.5)	(63.1, 93.7)	(−17.5, 7.5)		

Higher scores indicate better functioning. Negative change scores indicate lower scores (decreased functioning) after yoga. Negative effect sizes indicate that post-yoga scores were improved (higher).

**Table 6:** Average (standard deviation, SD) amount of time spent in physical activity pre- and post-yoga.

Intensity of Physical Activity	Pre-Yoga (*n* = 5)	Post-Yoga (*n* = 3)
Sedentary	11.0 (0.6) h	11.8 (0.8) h
	84 (1.4)% of day	86 (2.5)% of day
Light	1.5 (0.2) h	1.3 (0.2) h
	12 (1.4)% of day	10 (1.5)% of day
Moderate	23.0 (1.1) min	22.6 (0.1) min
	3 (0.3)% of day	3 (4.8)% of day
Vigorous	13.1 (4.3) min	12.3 (7.1) min
	2 (0.5)% of day	2 (0.9)% of day
